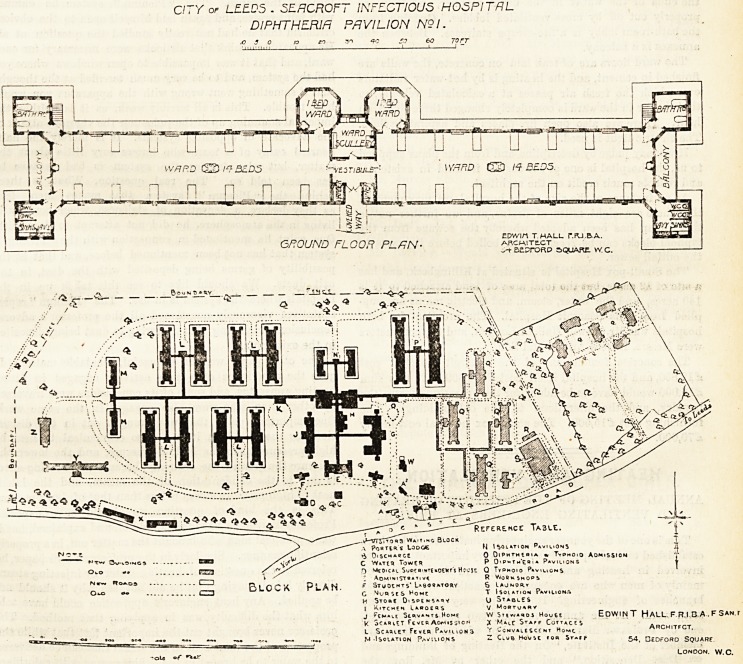# The Seacroft Hospital, Leeds

**Published:** 1905-07-01

**Authors:** 


### THE SEACROFT HOSPITAL, LEEDS.

IN the year 1892 the Corporation of the City of Leeds bought, for £8,000, an estate of 97 acres at Seacroft on the road leading to Tadcaster. The buildings erected at that time soon proved to be inadequate, and in 1898 a further amount of land was acquired for £21,250. Building operations were begun forthwith; and the hospital in its finished state was formally opened by the Lord Mayor of Leeds in the autumn of 1904.

The Fever Hospital stands on the south side of York Road and is about three miles from the centre of the City of Leeds.

branch road to each pavilion. Occupying a position almost central to the various blocks stands the administrative department and opposite to its main entrance but on the north side of the private road is the clock and water-tower. The clock has illuminated dials, and the tower is capable of storing 125 tons of water.

South of the administrative department runs a long corridor, at the east end of which are two isolation pavilions, and placed four on each side of the corridor are the scarlet fever pavilions. Near the west end of the main corridor are

The hospital consists of 42 separate blocks; and the aggregate length of these buildings in position from one end to the other is a quarter of a mile. The ground area occupied is 41 acres. Beneath this area is a worked-out coal mine; and to prevent local settlements it was considered desirable to place the separate blocks on rafts of cement concrete with steel girders incorporated in the concrete.

To the left of the main entrance of the grounds is the porter's lodge; and on the right are the stables, the mortuary, and the ambulance house. From the lodge a private road runs all round the buildings, and there is a two diphtheria blocks, and between these and the scarlet fever blocks are two additional pavilions for isolation Further westward are the typhoid fever blocks.

The medical superintendent's house is placed to the north of the private road, and opposite it is the students' laboratory. In addition to all these blocks there are operating theatre, discharging block, convalescent home, steward's house, clubhouse for the staff, and cottages for the male officials.

We give herewith a large scale drawing of the diphtheria block which may be taken as fairly representative of the other blocks in general arrangement. The covered way leads to the centre of this block at which point is the entrance-hall with the vestibule, the latter having on one side a linen-room and on the other side a larder. Adjoining the vestibule is the ward scullery, and on each side of this a single-bedded ward projects in septangular shape. By this plan good cross ventilation is obtained in the single-bedded rooms; and there is, perhaps, no better arrangement where the blocks are entirely distinct from each other, although there is the slight drawback that one bed in the dormitory cannot have a window on both sides. Each ward is about 103 feet long and 25 feet wide, and as there are 14 beds, each bed has about 185 square feet of superficial space. The sanitary annexes are placed at the ends of the wards in the usual manner, and they are properly cut off by cross-ventilated lobbies. Opening from the bath-room lobby is a fire-escape staircase. Between the annexes is a balcony.

The ward floors are of teak laid on concrete, the walls are finished in cement, and the heating is by hot-water radiators over which the fresh air passes at a calculated velocity, so that the air in the ward is completely changed three times an hour. There are also open fire-places and aspirating flues. The electric light is used.

If we may judge by description and from the plans supplied to us, the hospital is one of the best of its kind in existence, and reflects much credit on the architect.

As an instance of the size of the hospital, we may mention that there are eight miles of drains, and we further notice that a plan has been adopted whereby the sewage from the typhoid blocks can be received and boiled before it passes to the outfall sewer.

The Small-pox Hospital is situated at Killingbeck, and has a site of 12 acres, but the total area of land attached to it is 140 acres, and the water, steam, and electric mains are supplied from this Seacroft Hospital. The architect for both hospitals was Mr. Edwin Hall, of London, and the contractors were Messrs. Harold Arnold and Son.

The concrete foundations of the Seacroft Hospital cost £12,850, and the hospital itself cost £198,509. To this sum £15,000 would have to be added for the operating theatre, the laundry, and the alterations to the old buildings. The furnishing cost £15,500. The small-pox hospital cost nearly £70,000.

## Figures and Tables

**Figure f1:**